# Supplementary material for: Predicting the viability of beta-lactamase: How folding and binding free energies correlate with beta-lactamase fitness
Source: PLoS One. 2020 May 29;15(5):e0233509. doi: 10.1371/journal.pone.0233509 (PMC7259980; doi:10.1371/journal.pone.0233509)
Supplement: S1 File — Contains all supporting discussion regarding β-lactamase and methods, as well as two supporting tables and nine supporting figures. (ZIP) [file pone.0233509.s001.zip › Supplement.pdf]

Supplemental Information for “Predicting the Viability of Beta-Lactamase: How Folding and Binding Free Energies Correlate with Beta-Lactamase Fitness”

Jordan Yang[1], Nandita Naik[2], Jagdish Suresh Patel[3,4], Christopher S. Wylie[2], Wenze Gu[1], Jessie Huang[5], F. Marty Ytreberg[3,6], Mandar T. Naik[7], Daniel M. Weinreich[2], and Brenda M. Rubenstein[1*]

1 Department of Chemistry, Brown University, Providence, Rhode Island, United States of America
2 Department of Ecology and Evolutionary Biology, Brown University, Providence, Rhode Island, United States of America
3 Institute for Modeling Collaboration and Innovation, University of Idaho, Moscow, Idaho, United States of America
4 Department of Biological Sciences, University of Idaho, Moscow, Idaho, United States of America
5 Department of Chemistry, Wellesley College, Wellesley, Massachusetts, United States of America
6 Department of Physics, University of Idaho, Moscow, Idaho, United States of America
7 Department of Molecular Pharmacology, Physiology, and Biotechnology, Brown University, Providence, Rhode Island, United States of America

* brenda_rubenstein@brown.edu

Thermodynamic Biophysical Predictors

The primary thermodynamic measures employed by the community to predict fitness are the changes in the free energy of folding ($\Delta\Delta G_{fold} \equiv \Delta G_{fold,mutant} - \Delta G_{fold,wildtype}$, where $\Delta G_{fold,x} \equiv G_{native,x} - G_{unfolded,x}$ and x denotes the mutant or wild type proteins) and binding ($\Delta\Delta G_{bind} \equiv \Delta G_{bind,mutant} - \Delta G_{bind,wildtype}$, where $\Delta G_{bind,x} \equiv G_{bound,x} - G_{unbound,x}$). As follows from thermodynamics, the more negative the free energy of a system is, the more stable it is expected to be. These changes therefore indicate how much more stable mutant folding and binding are relative to wild type folding and binding. Free energies reflect a compromise between a molecule’s tendency to minimize its energy and maximize its entropy at a given temperature, and accordingly, all free energy estimates contain a mix of energetic and entropic contributions.

Beta-Lactamase and Its Relevant Biophysics

Beta-Lactamase

β-lactamases are enzymes produced by bacteria that provide resistance to a wide array of β-lactam antibiotics [1]. β-lactam antibotics contain a β-lactam ring in their molecular structures and inhibit cell wall synthesis. The β-lactamase resistance mechanism is based upon hydrolysis: β-lactamases hydrolyze the β-lactam ring present in all β-lactam antibiotics, which renders them inactive [2]. Among the types of β-lactamases found in Gram-negative bacteria, TEM-1, a class A, plasmid-encoded

broad spectrum serine β-lactamase, is the most prevalent, and accounts for 90% of *E. coli*'s β-lactam drug resistance [3, 4]. Wild type β-lactamases exhibit high levels of hydrolytic activity toward penicillins and mutant β-lactamases have gradually evolved to resist a much wider range of antibiotics, including several generations of cephalosporins [1, 5–7]. The emergence of different types of β-lactamase mutants therefore poses an enormous threat to modern-day antimicrobial therapies that has already had massive repercussions for the current drug market. Developing a fundamental understanding of the mechanism by which mutations are able to modify the structure and substrate specificity of TEM-1 β-lactamase is thus of tremendous practical importance to modern medicine.

Early single-site mutagenesis experiments revealed the effects of key single-point mutations of TEM-1 on enzyme stability and activity by characterizing the kinetics and protein abundance of the associated mutants. Many of these studies were the first to identify the most influential active site residues in β-lactamase catalysis, which include S70, the residue that first attacks the carbonyl of the lactam ring, K73, S130, K234, and A237, which assist the acylation [8–12], and E166, which activates the water that ultimately facilitates the release of the catalyzed substrate [13]. Beyond S130, the D131 and N132 residues that constitute the highly conserved 'SDN'-loop were additionally found to be nearly indispensable for proper enzyme function [9]. The substitution of N for S130 yielded a barely active protein; mutations of D130 into E and G were found to highly destabilize the protein; and replacement of N132 by A resulted in a dramatic decrease of enzyme activity [9]. Motivated by mutations observed in β-lactamases that have evolved to catalyze extended-spectrum cephalosporins [14], subsequent research has also characterized the Ω-loop region, consisting of residues 164-179 (residue 166 included), whose conformational flexibility is known to facilitate the coordination of the substrate [15, 16]. Residues 176, 178, and 179 were shown to participate in salt bridges key to maintaining the structure of the loop necessary for ampicillin catalysis [16]. Interestingly, recent studies have shown that, rather than being destabilizing as usual, certain, rare mutations, such as the M182T mutant, can stabilize β-lactamase against the deleterious effects of other mutations [17]. Moreover, somewhat paradoxically, mutating the S70 residue so critical to β-lactamase catalysis to glycine has been shown to relieve steric strain, thereby significantly improving enzyme stability [18]. Altogether, these single-site mutagenesis studies have provided researchers with a basic skeleton for understanding the amino acids critical to β-lactamase's function largely based upon kinetic analyses, but leave many possible, more subtle mutational influences to be uncovered and do not directly probe free energy changes.

In recent years, saturation mutagenesis [19] experiments combined with deep sequencing [20] have given the community the unprecedented ability to generate and analyze much more comprehensive libraries of mutations, for the first time granting researchers insights into how mutations' effects on fitness are distributed [21–23]. Some of the first saturation mutagenesis experiments on TEM-1 assessed relative mutant antibiotic resistance by competitively growing mutants in different concentrations of antibiotics [21, 22, 24]. These works were the first to estimate β-lactamase's distribution of fitness effects, which was found to be bimodal with most mutants leading to significant decreases in fitness, but with a handful remaining neutral/near-neutral [25]. Nevertheless, initial growth competition experiments often suffered from their limited ability to measure low-fitness alleles and thus characterize fitness valleys, while initial drug resistance assays suffered from low overall resolution. One particularly comprehensive mutagenesis study of TEM-1 distinctive in its use of genetic bandpass filters for β-lactamase activity to ensure that all point mutants of the TEM-1 β-lactamase gene and missense mutations of the TEM-1 protein were represented in its datasets was performed by Firnberg *et al.* [23]. By analyzing how fitness varied with free

energies of folding obtained using PyRosetta and the PoPMuSiC (see the Materials and Methods Section for a more complete discussion of PyRosetta and the Supplemental Information for a discussion of PoPMuSiC), these researchers illustrated that fitness precipitously declines for mutants for which $\Delta\Delta G_{fold}$ exceeds a few kcal/mol and that roughly 18% of the fitness according to PoPMuSiC can be explained by folding stability predictions according to linear models. The finding that fitness rapidly declines with $\Delta\Delta G_{fold}$ is consistent with a large body of work demonstrating that proteins possess stability thresholds beyond which they may be expected to be barely functional [26]. This study did not, however, attempt to further examine whether other thermodynamic or kinetic measures may be more predictive of fitness than folding free energies alone or directly obtain experimental folding free energies for use in its analyses.

Wylie Mutant Circular Dichroism Data at 25°C

Allele	Replicates	T_m°C	SEM	ΔH(kcal/mol)	SEM	ΔG (kcal/mol)	SEM
WT	2	56.80	0.15	-87.30	1.21	-8.41	0.15
A172P	2	57.05	0.10	-64.43	0.49	-6.25	0.03
A213G	3	57.02	0.33	-49.24	2.12	-4.77	0.16
D163Y	3	56.68	0.09	-37.74	0.45	-3.63	0.04
E212K	2	57.15	0.10	-107.62	1.20	-10.48	0.15
G144E	3	56.78	0.09	-41.82	0.79	-4.03	0.08
G218V	2	53.75	0.10	-49.38	0.18	-4.34	0.00
G283C	2	57.95	0.00	-64.00	0.09	-6.37	0.01
I142F	3	54.72	0.09	-61.48	0.47	-5.57	0.06
K234Q	3	57.38	0.09	-80.69	2.13	-7.91	0.20
L57H	3	56.62	0.09	-40.01	0.19	-3.84	0.02
L199F	3	54.85	0.00	-45.06	0.44	-4.10	0.04
R93S	3	56.98	0.07	-36.58	0.08	-3.54	0.01
R241H	3	58.58	0.07	-56.87	0.92	-5.76	0.08
R275G	3	56.65	0.17	-57.70	0.48	-5.54	0.04
S70G	3	61.55	0.15	-89.99	1.81	-9.83	0.17
A172P/G283C	3	57.98	0.19	-44.57	0.45	-4.44	0.03
A213G/L57H	3	59.38	0.15	-24.99	0.91	-2.58	0.10
D163Y/R93S	3	57.62	0.03	-38.86	0.53	-3.83	0.05
E212K/G218V	2	57.80	0.15	-68.66	1.13	-6.80	0.14
G144E/L199F	3	58.02	0.09	-40.54	0.49	-4.04	0.05
K234Q/R241H	3	59.15	0.00	-56.09	1.29	-5.76	0.13

Table S1. Table of Wylie mutants and their respective thermodynamic data according to circular dichroism experiments performed at 25°C.

Wylie Mutant Data Set Computational Folding Free Energy Predictions

We acquired $\Delta\Delta G_{fold}$ data on the Wylie mutant data set using FoldX with and without relaxing wild type conformations via molecular dynamics (MD) before mutations were introduced. Here, for completeness, we compare and contrast our FoldX results with the MD+FoldX and PyRosetta results discussed in the main text.

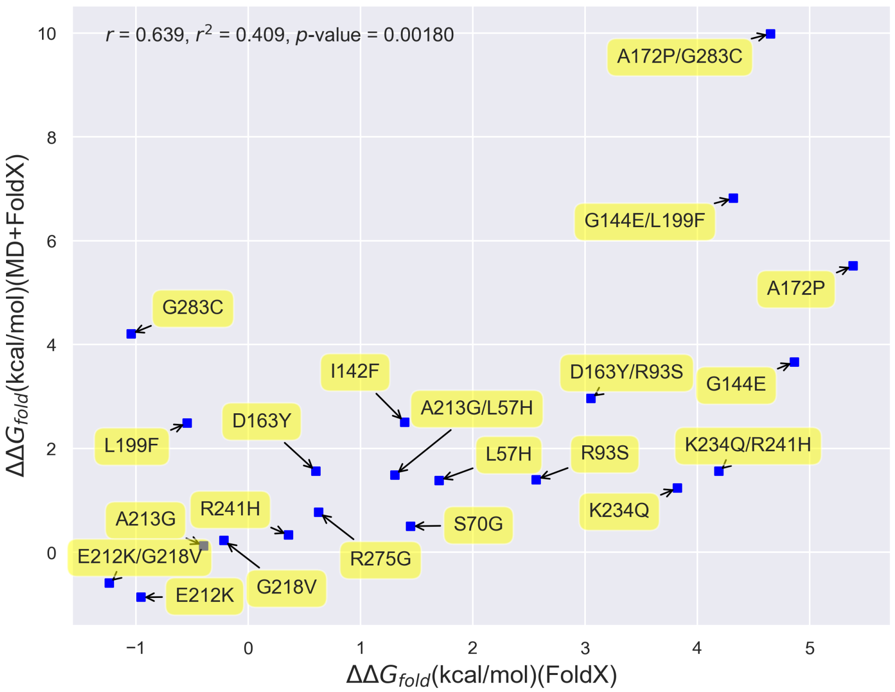

Fig S1. Comparison between FoldX free energy of folding results without using molecular dynamics simulations to relax wild type protein conformations into which mutations are introduced and MD+FoldX results with relaxation for the Wylie mutant data set. As may be expected and is exemplified by the plot, unrelaxed FoldX calculations tend to overestimate folding free energies compared to their relaxed counterparts. Only G238C, G283C/A172P, and G144E/L199F significantly deviate from a linear trend.

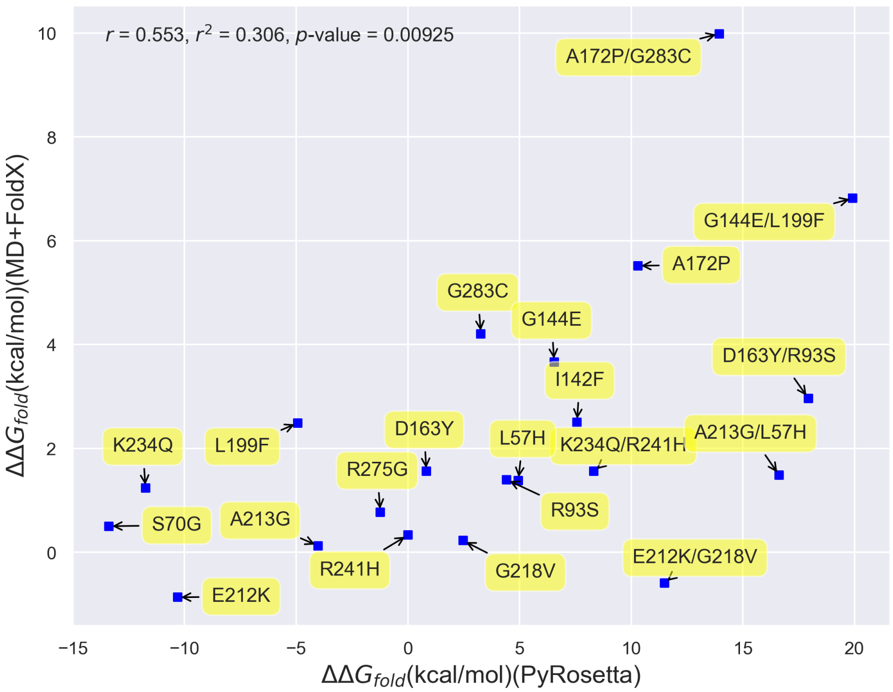

Fig S2. Comparison between MD+FoldX and PyRosetta results for the folding free energies of the Wylie mutant data set. PyRosetta tends to overestimate folding free energies relative to MD+FoldX results.

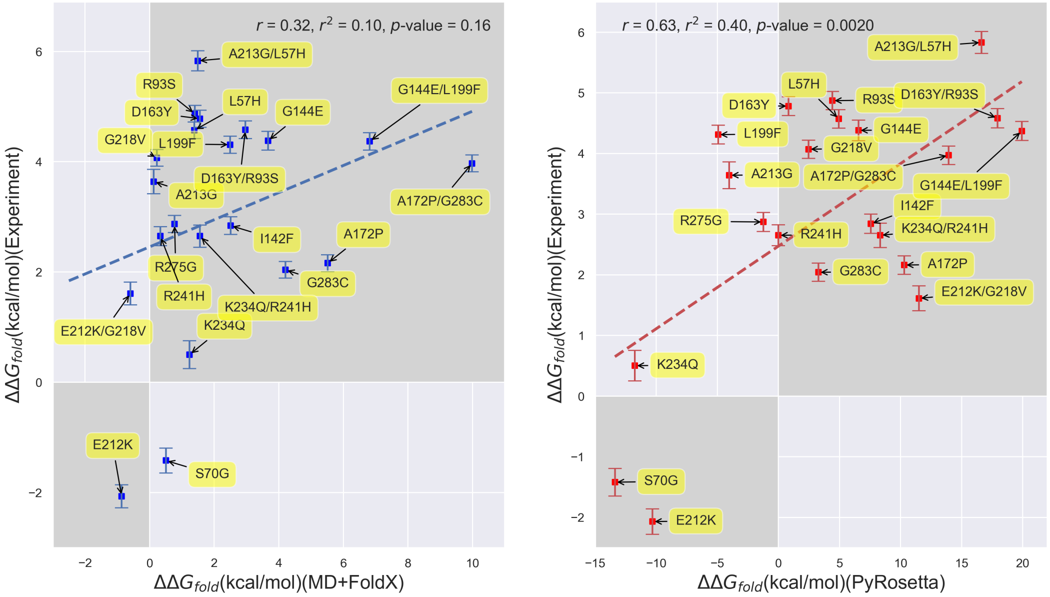

Fig S3. Scatterplots depicting the correlation between experimentally- and computationally-determined β-lactamase TEM-1 free energies of folding for multiply-mutated proteins and their constituent single mutants. **(Left)** Correlation based upon MD+FoldX free energy predictions. **(Right)** Correlation based upon PyRosetta free energy predictions. The labels on the points name the mutations involved. The shaded regions delineate the first and third quadrants of the plot, which contain mutants whose free energies are of the same sign according to both experiment and computation.

Free Energies of Double Mutants and Their Constituent Single Mutants						
Amino Acid # (Ambler)	$\Delta\Delta G_{fold}$ (MD+FoldX)	$\Delta\Delta G_{fold}$ (MD+FoldX) [Mut A + Mut B]	$\Delta\Delta G_{fold}$ (PyRosetta)	$\Delta\Delta G_{fold}$ (PyRosetta) [Mut A+Mut B]	$\Delta\Delta G_{fold}$ (Exp)	$\Delta\Delta G_{fold}$ (Exp) [Mut A+Mut B]
**Single Mutants**						
A172P	5.519		10.302		2.16 ± 0.15	
A213G	0.126		-4.018		3.64 ± 0.22	
D163Y	1.560		0.829		4.78 ± 0.15	
E212K	-0.866		-10.306		-2.07 ± 0.21	
G144E	3.666		6.555		4.38 ± 0.17	
G218V	0.277		2.471		4.07 ± 0.15	
G283C	4.205		3.274		2.04 ± 0.15	
K234Q	1.236		-11.748		0.50 ± 0.25	
L57H	1.385		4.951		4.57 ± 0.15	
L199F	2.490		-4.933		4.31 ± 0.16	
R93S	1.400		4.419		4.87 ± 0.15	
R241H	0.332		0.0037		2.65 ± 0.17	
**Double Mutants**						
A172P/G283C	9.987	9.724	13.945	13.576	3.97 ± 0.15	4.20 ± 0.21
A213G/L57H	1.489	1.511	16.626	0.933	5.83 ± 0.18	8.21 ± 0.27
D163Y/R93S	2.966	2.96	17.937	5.248	4.58 ± 0.16	9.65 ± 0.22
E212K/G218V	-0.591	-0.639	11.499	-7.835	1.61 ± 0.21	2.00 ± 0.26
G144E/L199F	6.817	6.156	19.927	1.622	4.37 ± 0.16	8.69 ± 0.23
K234Q/R241H	1.559	1.568	8.335	-11.744	2.65 ± 0.20	3.16 ± 0.31

Fig S4. Table of MD+FoldX, PyRosetta, and experimental $\Delta\Delta G_{fold}$ values for double mutants and their constituent singles. For the double mutants, the directly computed/measured $\Delta\Delta G_{fold}$ values and those values obtained by adding the $\Delta\Delta G_{fold}$ values of the related constituent singles (denoted as $\Delta\Delta G_{fold}$ (Method X)[Mut A + MutB]) are reported for each method for comparison. All values are given in units of kcal/mol. All mutations are named according to the Ambler notation as described in the main text; double mutants are reported with a slash in between each individual mutant's designation. The error bars that accompany the experimental values are propagated standard errors on the mean computed based upon mutant ΔG values measured in triplicate.

Properties of Wylie Single Mutants			
Mutant	Change in Hydrophobicity	Solvent-Exposed?	In Which Secondary Structure?
A172P	Decrease	Yes	Coil
A213G	Increase	Yes	Coil
D163Y	Increase	Yes	Coil
E212K	Increase	Yes	Helix
G144E	Decrease	Yes	Coil
G218V	Increase	Yes	Coil
G283C	Increase	No	Helix
I142F	Increase	Yes	Helix
K234Q	Increase	Yes	Sheet
L57H	Decrease	Yes	Sheet
L199F	Increase	Yes	Coil
R93S	Increase	Yes	Coil
R241H	Increase	Yes	Coil
R275G	Increase	Yes	Helix
S70G	Increase	Yes	Helix

Fig S5. Table of the properties of the Wylie single mutants studied in this work. Note that, while these mutants reside in a variety of secondary structures, all but one is solvent-exposed and the overwhelming majority substitute a more hydrophobic residue for a less hydrophobic wild type residue.

Correlations Involving Ampicillin Minimum Inhibitory Concentrations

To assess how well our free energy data predicted β-lactamase viability, we compared our results against minimum inhibitory concentrations (MIC) of β-lactamase acquired for the Wylie mutant data set as well as fitness data determined by Firnberg *et al.* [23]. Our procedure for ascertaining MIC values is detailed below. Further details may be found in Weinreich *et al.* [27].

Determination of Wylie Mutant Data Set Minimum Inhibitory Concentrations

The Wylie mutants were constructed using the QuikChange protocol (Stratagene, San Diego, CA) as described in Weinreich *et al.* (2006) [27]. The specific single missense mutants presented in Table 1 of the main text were chosen at random subject only to the condition that they lie at least 6 Å outside of the TEM-1 active site according to PDB record 1BTL and therefore should negligibly affect binding. The pairs of mutations in the double mutants shown were chosen at random from among the single mutants. To construct the Wylie data set of β-lactamase alleles, mutations were introduced into the AmpR locus of the cloning vector pBR322 (New England Biolabs, Beverly, MA, identical to TEMwt) using the Qiagen QuikChange kit (Stratagene, La Jolla, CA; mutagenesis primer sequences available upon request), transformed into the chemically competent *E. coli* strain DH5α, and sequenced to confirm identity on an ABI 3100 (Applied Biosystems, Foster City, CA; sequencing primers available upon request). The minimum inhibitory concentration (MIC) of each transformant and of control strains were simultaneously assayed in triplicate by the broth microdilution method. Briefly, a series of 39 2-fold dilutions of ampicillin (Sigma-Aldrich, St. Louis, MO) from 8196.0 μg/ml to 0.015625 μg/ml were prepared in Difco Muller-Hinton broth (BD, Franklin Lakes, NJ), together with a positive control of Muller-Hinton broth lacking the drug. Bacterial overnight stocks were diluted to $\sim 10^6$ cfu/mL in Muller-Hinton broth, and 150 μl of each was combined with 150 μl of each drug dilution in covered sterile 96- well flat-bottom polystyrene plates (VWR, West Chester, PA). These were placed in plastic bags to limit evaporation and incubated for 20 hours at 35°C. The MIC was recorded as the lowest drug concentration that blocked any visual evidence of bacterial growth. MIC values for each allele were identical across replicates in all but six cases. We then repeated the assay in six-fold replicate for 12 alleles: those six that exhibited variation and six others matched to these by similar MIC values. These results confirmed that this low level of variation was typical for the MIC assay in our hands and also that variation in MIC was not allele-specific.

Correlation Between MIC Data and Firnberg Fitness Data

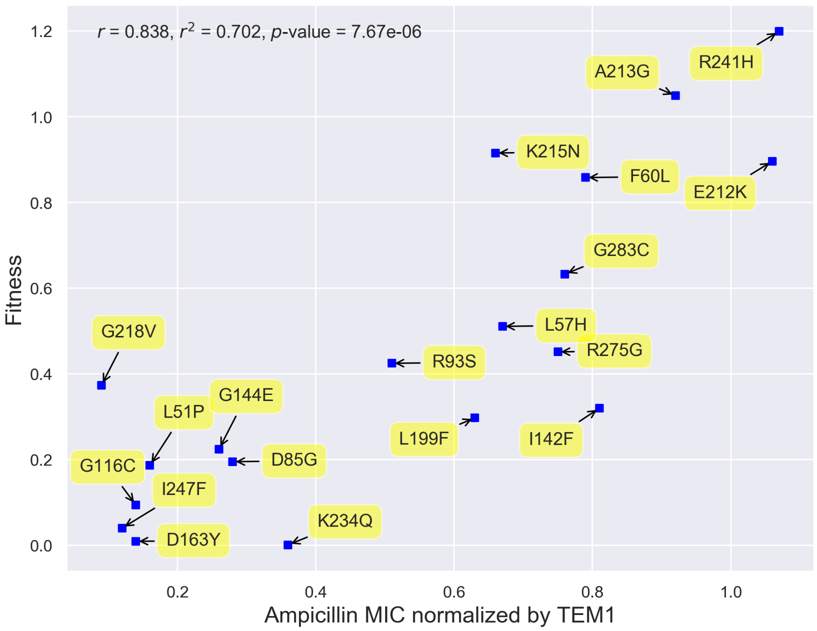

Fig S6. Comparison between the fitness of the β-lactamase mutants as reported by Firnberg [23] and our ampicillin minimum inhibitory concentrations. The strong correlation between these measures of fitness gives credence to utilizing the Firnberg data sets in our analyses of how predictive free energy changes are of fitness.

Correlation Between Wylie MIC Data and Folding Free Energies

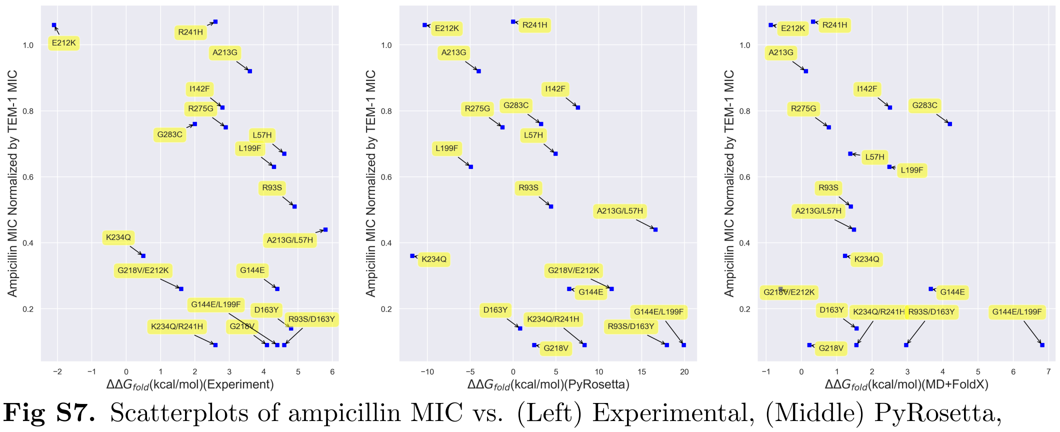

Fig S7. Scatterplots of ampicillin MIC vs. (Left) Experimental, (Middle) PyRosetta, and (Right) MD+FoldX folding free energy changes for the Wylie mutant data set. No clear trends emerge between MIC and experimental folding free energies, while plotting MIC against computational folding free energies suggests weak negative correlations between these quantities.

Firnberg Mutant Dataset Folding and Binding Free Energy Predictions

Overview of the PoPMuSiC Algorithm for Folding Free Energy Predictions

PoPMuSiC predictions of $\Delta\Delta G_{fold}$ values were determined online at https://soft.dezyme.com/query/create/pop [28] using 1xpb. PoPMuSiC is a web server that allows fast and accurate predictions of the thermodynamic stability changes caused by single site mutations in globular proteins using a linear combination of 13 statistical potentials, including two terms that depend on the volume of the wild type and mutant amino acids and a term that accounts for solvent accessibility. This web server distinguishes itself from other similar software prediction tools by its high computational speed as well as by its ability to perform a systematic evaluation of all possible mutations in a globular protein. It is currently publicized as the only computational prediction tool that allows a systematic scan of all possible mutations of a medium size globular protein in less than a minute via the implementation of a single, simple, web-based query.

Categorization of Figure 7 Mutants According to Fitness and Computational Predictions

	$\Delta\Delta G_{fold} < 5$ **kcal/mol**	$\Delta\Delta G_{fold} > 5$ **kcal/mol**
**Fitness** > 0.5	2175 (True Positives)	22 (False Negatives)
**Fitness** < 0.5	2079 (False Positives)	506 (True Negatives)

Table S2. Number of true positive, false positive, false negative, and true negative β-lactamase mutants according to computational predictions and experimental fitness data.

Non-Linear Fits to Firnberg Fitness vs. Folding and Binding Free Energy Data

In order to analyze our fitness data beyond simple linear fits that cannot account for mutants with large folding and binding free energies, we also fit the data in main text Figures 7 and 9 with the non-linear functional forms with the smallest sum of squares errors from a selection of functions provided by Zunzun.com (http://zunzun.com/). While even better fits could potentially be achieved using other functions, these nonlinear functional forms grant one insight into the improvements that may be gained by going beyond linear fits and account for the rapid decline in fitness observed when plotted against either increasing folding or binding data.

Firnberg Mutant Dataset Binding Free Energy Predictions

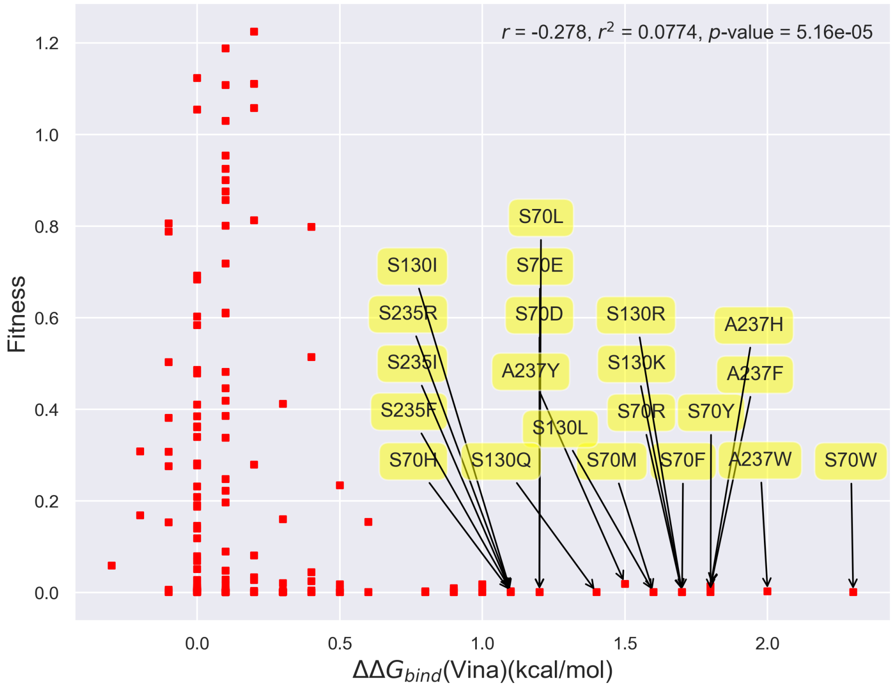

Fig S8. Scatterplot of fitness vs. binding free energies for single mutants within 8 Å of the active site (S70 alpha carbon). Many of the mutants with the most positive binding free energies are located in the active site, as described in the Introduction.

Top Pose of Ampicillin Docked to Beta-Lactamase

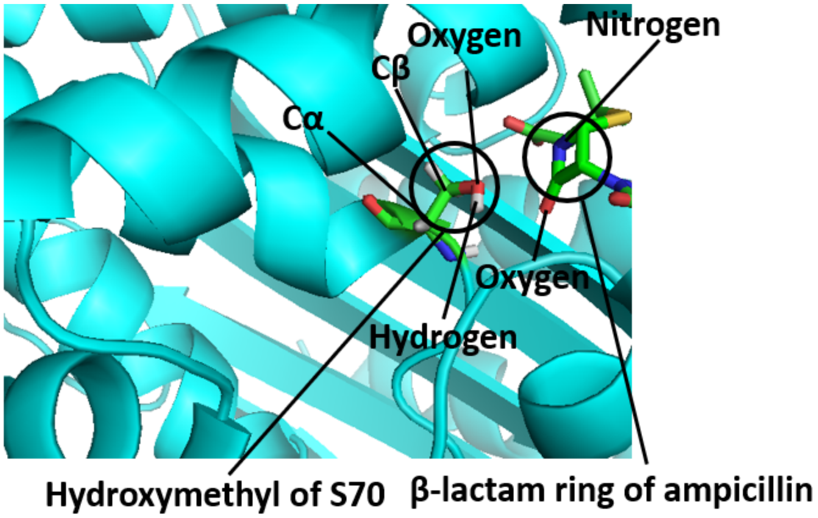

Fig S9. A top docked pose of ampicillin generated via AutoDock Vina in the relaxed wild type β-lactamase conformation, in which the oxygen of the β-lactam ring is interacting with the hydroxyl group of S70. The distance between the oxygen of the β-lactam ring and the oxygen of the hydroxyl group is 3.033 Å, and the distance between the oxygen of the β-lactam ring and the hydrogen of the hydroxyl group is 3.281 Å.

References

1. Morin RB, Gorman M. The Biology of B-Lactam Antibiotics. Elsevier; 2014.
2. Page MI, Proctor P. Mechanism of beta-lactam ring opening in cephalosporins. Journal of the American Chemical Society. 1984;106(13):3820–3825.
3. Escobar WA, Tan AK, Fink AL. Site-directed mutagenesis of beta-lactamase leading to accumulation of a catalytic intermediate. Biochemistry. 1991;30(44):10783–10787.
4. Sutcliffe JG. Nucleotide sequence of the ampicillin resistance gene of Escherichia coli plasmid pBR322. Proceedings of the National Academy of Sciences. 1978;75(8):3737–3741.
5. Ambler R, Coulson A, Frere JM, Ghuysen JM, Joris B, Forsman M, et al. A standard numbering scheme for the class A beta-lactamases. Biochemical Journal. 1991;276(Pt 1):269.
6. Huletsky A, Knox J, Levesque R. Role of Ser-238 and Lys-240 in the hydrolysis of third-generation cephalosporins by SHV-type beta-lactamases probed by site-directed mutagenesis and three-dimensional modeling. Journal of biological chemistry. 1993;268(5):3690–3697.
7. Sowek JA, Singer SB, Ohringer S, Malley MF, Dougherty TJ, Gougoutas JZ, et al. Substitution of lysine at position 104 or 240 of TEM-1pTZ18R beta-lactamase enhances the effect of serine-164 substitution on hydrolysis or affinity for cephalosporins and the monobactam aztreonam. Biochemistry. 1991;30(13):3179–3188.

8. Gibson RM, Christensen H, Waley SG. Site-directed mutagenesis of beta-lactamase I. Single and double mutants of Glu-166 and Lys-73. Biochemical Journal;(3):613–619.

9. Jacob F, Joris B, Lepage S, Dusart J, Frère JM. Role of the conserved amino acids of the 'SDN' loop (Ser^{130}, Asp^{131} and Asn^{132}) in a class A β-lactamase studied by site-directed mutagenesis. Biochemical Journal. 1990;271(2):399–406.

10. Ellerby LM, Escobar WA, Fink AL, Mitchinson C, Wells JA. The role of lysine-234 in beta-lactamase catalysis probed by site-directed mutagenesis. Biochemistry. 1990;29(24):5797–5806.

11. Damblon C, Raquet X, Lian LY, Lamotte-Brasseur J, Fonze E, Charlier P, et al. The catalytic mechanism of beta-lactamases: NMR titration of an active-site lysine residue of the TEM-1 enzyme. Proceedings of the National Academy of Sciences. 1996;93(5):1747–1752.

12. Matagne A, Frere JM. Contribution of mutant analysis to the understanding of enzyme catalysis: The case of class A beta-lactamases. Biochimica et Biophysica Acta (BBA) - Protein Structure and Molecular Enzymology. 1995;1246(2):109 – 127.

13. Leung YC, Robinson CV, Aplin RT, Waley SG. Site-directed mutagenesis of beta-lactamase I: role of Glu-166. Biochemical Journal;(Pt 3):671–678.

14. Huletsky A, Knox J, Levesque R. Role of Ser-238 and Lys-240 in the hydrolysis of third-generation cephalosporins by SHV-type beta-lactamases probed by site-directed mutagenesis and three-dimensional modeling. Journal of Biological Chemistry. 1993;268:3690–3697.

15. Palzkill T, Le QQ, Venkatachalam KV, LaRocco M, Ocera H. Evolution of antibiotic resistance: several different amino acid substitutions in an active site loop alter the substrate profile of beta-lactamase. Molecular Microbiology. 1994;12(2):217–229.

16. Petrosino JF, Palzkill T. Systematic mutagenesis of the active site omega loop of TEM-1 beta-lactamase. Journal of bacteriology. 1996;178(7):1821–1828.

17. Sideraki V, Huang W, Palzkill T, Gilbert HF. A secondary drug resistance mutation of TEM-1 beta-lactamase that suppresses misfolding and aggregation. Proceedings of the National Academy of Sciences. 2001;98(1):283–288. doi:10.1073/pnas.98.1.283.

18. Stojanoski V, Adamski CJ, Hu L, Mehta SC, Sankaran B, Zwart P, et al. Removal of the side chain at the active-site serine by a glycine substitution increases the stability of a wide range of serine beta-lactamases by relieving steric strain. Biochemistry. 2016;55(17):2479–2490.

19. Siloto RMP, Weselake RJ. Site saturation mutagenesis: Methods and applications in protein engineering. Biocatalysis and Agricultural Biotechnology. 2012;1(3):181 – 189.

20. Fowler DM, Araya CL, Fleishman SJ, Kellogg EH, Stephany JJ, Baker D, et al. High-resolution mapping of protein sequence-function relationships. Nature Methods. 2010;7:741–746.

21. Jacquier H, Birgy A, Le Nagard H, Mechulam Y, Schmitt E, Glodt J, et al. Capturing the mutational landscape of the beta-lactamase TEM-1. Proceedings of the National Academy of Sciences. 2013;110(32):13067–13072.

22. Deng Z, Huang W, Bakkalbasi E, Brown NG, Adamski CJ, Rice K, et al. Deep sequencing of systematic combinatorial libraries reveals β-lactamase sequence constraints at high resolution. Journal of molecular biology. 2012;424(3-4):150–167.

23. Firnberg E, Labonte JW, Gray JJ, Ostermeier M. A comprehensive, high-resolution map of a Gene's fitness landscape. Molecular Biology and Evolution. 2014;31(6):1581–1592.

24. Bershtein S, Segal M, Bekerman R, Tokuriki N, Tawfik DS. Robustness–epistasis link shapes the fitness landscape of a randomly drifting protein. Nature. 2006;444(7121):929–932.

25. Stiffler MA, Hekstra DR, Ranganathan R. Evolvability as a function of purifying selection in TEM-1 beta-lactamase. Cell. 2015;160(5):882 – 892.

26. Tokuriki N, Tawfik DS. Stability effects of mutations and protein evolvability. Current Opinion in Structural Biology. 2009;19(5):596–604.

27. Weinreich DM, Delaney NF, DePristo MA, Hartl DL. Darwinian evolution can follow only very few mutational paths to fitter proteins. Science. 2006;312(5770):111–114.

28. Dehouck Y, Kwasigroch JM, Gilis D, Rooman M. PoPMuSiC 2.1: a web server for the estimation of protein stability changes upon mutation and sequence optimality. BMC bioinformatics. 2011;12(1):151.
